# Management of chest pain: a prospective study from Norwegian out-of-hours primary care

**DOI:** 10.1186/1471-2296-15-51

**Published:** 2014-03-24

**Authors:** Robert Anders Burman, Erik Zakariassen, Steinar Hunskaar

**Affiliations:** 1 National Centre for Emergency Primary Health Care, Uni Research Health, Kalfarveien 31, 5018 Bergen, Norway; 2 Department of Global Public Health and Primary Care, University of Bergen, Post box 7804, 5020 Bergen, Norway; 3 Department of Research, Norwegian Air Ambulance Foundation, Post box 94, 1441 Drøbak, Norway

**Keywords:** Chest pain, Primary care, Out-of-hours, ECG, Severity of illness

## Abstract

**Background:**

Chest pain is a common diagnostic challenge in primary care and diagnostic measures are often aimed at confirming or ruling out acute ischaemic heart disease. The aim of this study was to investigate management of patients with chest pain out-of-hours, including the use of ECG and laboratory tests, assessment of severity of illness, and the physicians’ decisions on treatment and admittance to hospital.

**Methods:**

Data were registered prospectively from four Norwegian casualty clinics. Data from structured telephone interviews with 100 physicians shortly after a consultation with a patient presenting at the casualty clinic with “chest pain” were analysed.

**Results:**

A total of 832 patients with chest pain were registered. The first 100 patients (corresponding doctor-patient pairs) were included in the study according to the predefined inclusion criteria. Median age of included patients was 46 years, men constituted 58%. An ECG was taken in 92 of the patients. Of the 24 patients categorised to acute level of response, 15 had a NACA-score indicating a potentially or definitely life-threatening medical situation. 50 of the patients were admitted to a hospital for further management, of which 43 were thought to have ischaemic heart disease. Musculoskeletal pain was the second most common cause of pain (n = 22). Otherwise the patients were thought to have a variety of conditions, most of them managed at a primary care level.

**Conclusions:**

Patients with chest pain presenting at out-of-hours services in Norway are investigated for acute heart disease, but less than half are admitted to hospital for probable acute coronary syndrome, and only a minority is given emergency treatment for acute coronary syndrome. A wide variety of other diagnoses are suggested by the doctors for patients presenting with chest pain. Deciding the appropriate level of response for such patients is a difficult task, and both *over*- and *under*-triage probably occur in out-of-hours primary care.

## Background

Chest pain is a common diagnostic challenge in primary care for both general practitioners (GPs) during day time surgery hours and in casualty clinics out-of-hours [1-4]. Diagnostic measures are often aimed at confirming or ruling out acute ischaemic heart disease (IHD). However, in primary care less serious conditions frequently occur in patients with chest pain, such as musculoskeletal pain, dyspepsia and psychogenic disorders [5-8]. Previous research has shown that approximately only 5% of all patients with chest pain presenting in general practice have acute IHD; while as many as 50% may have myalgia and chest wall syndromes [7,9]. In emergency consultations out-of-hours, either at a casualty clinic or an urgent house call by a GP, the prevalence of acute IHD may still be as low as 15% [9].

In Norway, patients with chest pain in need of acute medical assistance are encouraged to call the national three digits emergency telephone number ″113″. Still, many patients with chest pain choose to contact their GP directly, or the local casualty clinic out-of-hours. A recent study from Norway showed that patients with chest pain constituted 21% of all medical emergencies outside hospitals. The study also revealed that most of the patients were not as ill as initially assessed at the emergency medical communication centres, pointing to the challenges in deciding the appropriate level of response in patients with chest pain outside hospitals [3].

Diagnosing chest pain in primary care is a complex task. Previous studies have confirmed the importance of a thorough patient history on sensation of pain (type, duration, localisation etc.) and concomitant symptoms when diagnosing acute IHD [8,9]. Still, without cardiac markers (i.e. troponin) and more advanced diagnostic tools, many patients will be admitted to a hospital for further testing and treatment. Electrocardiogram (ECG) is a crucial diagnostic tool for patients with chest pain, but although ECG is a diagnostic test with high specificity, the sensitivity of the test in clinical practice is low, making it difficult to rule out IHD based on ECG alone [10,11].

In a hospital setting, patients with chest pain of suspected cardiac origin are often diagnosed and treated according to specific guidelines and to some extent clinical decision rules. The pre-test probability of IHD is greater (“high prevalence setting”) than in primary care (“low prevalence setting”) and diagnostic tools are readily available to make more definitive diagnoses. Previous studies have shown that Norwegian out-of-hours services generally are well-equipped with laboratory and diagnostic tools, but the selection of tests are mainly adapted to a primary care setting [12,13]. One study reported that ECGs were taken in 4% of all consultations [12]. Another study showed that 99% of all Norwegian casualty clinics had an ECG-device, while only 6% of the casualty clinics could measure d-dimer and/or troponin locally [13].

Little is still known about the management of chest pain in Norwegian out-of-hours primary care. No research exists on the use of diagnostic tools; how patients with chest pain are treated; or how many patients that end up being admitted to a hospital.

The aim of this study was to investigate the use of diagnostic tools and treatment of choice in patients with acute chest pain out-of-hours in Norwegian primary care. We registered the use of ECG and other laboratory tests, assessed the severity of illness, and also the physicians’ decisions on treatment and admittance strategies.

## Methods

Four Norwegian casualty clinics, located at Sotra, Haugesund, Drammen and Kristiansand, were involved in the study. The casualty clinics were chosen according to strategic sampling to cover both rural, suburban and urban districts, and to include both larger and smaller casualty clinics. Data were collected prospectively from February to July 2012.

Data in the analyses come from structured telephone interviews with 100 physicians shortly after a consultation with a patient presenting at the casualty clinic with “chest pain” as his or her main symptom. Each physician could only be interviewed once, and the casualty clinics continued registration of patients until the predefined number of 100 unique physicians with 100 corresponding patients had been included. The number of included physicians and patients were chosen to ensure the possibility of interviewing all physicians shortly after the consultation, and to ensure a large enough sample to perform sub group analyses. The patients were registered prospectively by the nurses at the cooperating casualty clinics. All patients with “chest pain” or equivalent symptoms, independent of the probable cause of complaint, were registered with a unique identification number in a patient log. The variables recorded were consultation date and time, name, birth date, sex, age of the patient, response level and name and telephone number of the physician who treated the patient. Equivalent symptoms to chest pain included “tightness in chest”, “retrosternal pain” and “chest discomfort”. Patients with symptoms suggestive of mastitis were excluded. One of the authors (RAB) had daily contact with the four casualty clinics, gathering all registered patients and variables, excluding patient name and date of birth to achieve anonymous data collection. Before patient inclusion started, all nurses and physicians at the cooperating casualty clinics were informed of the study through information meetings and distribution of the inclusion criteria and the study protocol. Oral consent was obtained from the physicians at the beginning of the interview. To ensure anonymous data collection, the physicians were explicitly asked to not disclose the patient’s name and/or date of birth. If a physician could not be reached by telephone, and interviewed, within 2 days after the consultation, he or she was excluded from participation, to reduce recall bias. The variable “level of response” was set by the nurses at the casualty clinic using the Norwegian Index of Medical Emergencies [14]. The Index categorises clinical symptoms, findings and incidents into a red, yellow and green criteria based section, correlating to the appropriate level of response. Red colour is defined as an “acute” response, with the highest priority. Yellow colour is defined as an “urgent” response, with a high, but lower priority, where the patient should be examined as soon as the doctor-on call is available. Green colour is defined as a “non-urgent” response, with the lowest priority.

The questionnaire used in the telephone interview had two parts, where the first part consisted of questions related to the patient they just had treated, including diagnostic measures (use of ECG and laboratory analyses) and choice of treatment. Severity of illness was set by the physicians using The National Committee on Aeronautics (NACA) Score System [15]. In the NACA system, the patient’s status is classified from 0 to 7, zero indicating no disease or injury, while seven indicates the patient being dead (Table 1). NACA score was categorised in the analyses as NACA 0–1 (patient with either no symptoms/injuries or not in need of medical treatment), NACA 2–3 (patient in need of medical help, where value 3 indicates need of hospitalisation, but still not a life-threatening situation), NACA 4–6 (4 is a potentially, and 5 and 6 are definitely, life-threatening medical situations) and NACA 7 (dead person). The physicians were also asked to state what he or she judged to be the most probable cause of the symptoms. Finally, if the patient was admitted to a hospital, referred to a GP or a specialist, or got final treatment at the casualty clinic. The remainder of the questions focused on the individual physician’s approach to diagnosing patients with chest pain and reasons for hospital admission in general. These data will be described elsewhere.

**Table 1:** National committee on Aeronautics (NACA) score, used to decide severity of illness

**Score level**	**Patient status**
NACA 0	No injury or illness
NACA 1	Not acute life-threatening disease or injury
NACA 2	Acute intervention not necessary, further diagnostic studies needed
NACA 3	Severe, but not life threatening disease or injury; acute intervention necessary
NACA 4	Development of vital (life threatening) danger possible
NACA 5	Acute vital (life threatening) danger
NACA 6	Acute cardiac or respiratory arrest
NACA 7	Death

### Statistics

IBM Statistical Package for the Social Sciences (IBM SPSS version 20) was used for statistical analyses. Standard univariate statistics were used to describe the material, including mean and median. Student’s t-test was used to compare mean age between all registered patients and the included study patients. For other comparisons the Pearson Chi-Square test was used. A p-value of < 0.05 was considered statistically significant.

### Ethics

The study was given approval by the Regional Committee for Medical and Health Research Ethics (REC West) before inclusion started (Reference number 2010/1499-10).

## Results

**Figure 1:**
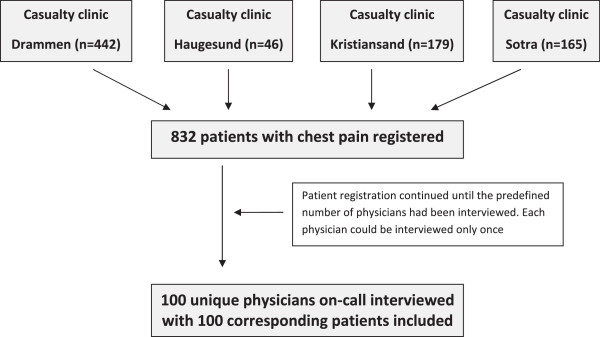
Flow chart of registration of patients and the inclusion process.

A total of 832 patients with chest pain were registered at the four participating casualty clinics, of which 100 patients with corresponding structured telephone interviews with the physician on-call, were included in the study (Figure 1). All but one of the contacted physicians gave consent and wanted to participate in the study. The physicians included in the study were made up by 67 GPs and 33 other (11 interns in GP-practice, the rest hospital-based residents).

Table 2 shows a comparison between the registered patients not included (n = 732) and the included study patients (n = 100) with regard to mean age, age groups, sex and level of emergency response. In the study population (n = 100) the patient’s age ranged from 18 to 92 years (median age 46 years), 58% males with a median age of 45 years, and 42% females with median age 51 years. The two groups did not differ in any of the variables stated, except mean age, the study patients were about 5 years younger (p < 0.05).

**Table 2:** Comparison between all registered patients and the included study patients

	**Registered patients, not included (N = 732)**	**Included study patients (n = 100)**	**P-value**
**Age, years (mean)**	55	50	*0.016*
**Age categories, distribution**			*0.086*
**18-35 years**	17%	23%	
**36-50 years**	26%	33%	
**51-65 years**	24%	23%	
**66-80 years**	21%	15%	
**>80 years**	12%	6%	
**Sex (female)**	46%	42%	*0.494*
**Level of response, distribution**			*0.451*
***Red***	19%	24%	
***Yellow***	68%	66%	
***Green***	13%	10%	

Level of response was set using the Norwegian Index of Medical Emergencies.

Table 3 describes the level of response set by the nurse using the Index compared to severity of illness (NACA score) judged by the physicians, and the use of supplemental diagnostic tools such as ECG and other laboratory tests. Red response was set in 24 patients, 66 were given yellow response, the remainder 10 green response. An ECG was taken in 92 of the patients. Of the eight patients where an ECG was not taken, four were given response level “yellow”, and the last four “green response”. 52% (n = 48) of the ECGs were ordered by the nurse at the casualty clinic, in 24% (n = 22) the physician ordered the test, and in 15% (n = 14) the ECG was taken in the ambulance. In 8% (n = 7) an ECG was taken both in the ambulance and at the casualty clinic. Other laboratory tests were taken in 57% of the patients. Oxygen-saturation (n = 44) and C-reactive protein (n = 29) were the tests most often used, while d-dimer (n = 3) and other blood tests (glucose and haematology) were rarely done. 63% (n = 15) of the patients with a NACA-score indicating a potentially or definitely life-threatening medical situation (NACA 4–6) were categorised to “red response”, leaving 11 patients (37%) with a lower response level (yellow or green). Nine of the ten patients with “green response” were not in a life-threatening situation, leaving one patient with a NACA-score indicating immediate need of help.

**Table 3:** The use of diagnostic tools and severity of illness (NACA-score) by level of response (Norwegian Medical Index) for the included 100 patients

	**Level of response**			
	**Red**	**Yellow**	**Green**	**Total**
**ECG taken?**				
Yes	24	62	6	**92**
No	0	4	4	**8**
Total	24	66	10	**100**
**Who ordered the ECG?**				
Ambulance	9	5	0	**14**
Nurse at the casualty clinic	10	35	3	**48**
Physician at the casualty clinic	3	16	3	**22**
Both ambulance and casualty clinic	2	5	0	**7**
Unknown	0	1	0	**1**
Total	24	62	6	**92**
**Any laboratory test taken?**				
Yes	15	37	5	**57**
No	9	29	5	**43**
Total	24	66	10	**100**
**Laboratory test (more than one possible)**				
Oxygen-saturation	13	29	2	**44**
C-reactive protein	2	23	4	**29**
D-dimer	0	3	0	**3**
Other blood tests (glucose, haematology)	1	5	0	**6**
**Severity of illness; 0 = no disease, 7 = dead**				
NACA 0	1	0	0	**1**
NACA 1	1	18	5	**24**
NACA 2	4	18	3	**25**
NACA 3	3	20	1	**24**
NACA 4	10	9	1	**20**
NACA 5	4	1	0	**5**
NACA 6	1	0	0	**1**
NACA 7	0	0	0	**0**
Total	24	66	10	**100**

Medication was prescribed or given at the casualty clinic in 43% of the patients. Of the 43 patients, sublingual nitro-glycerine (67%, n = 29) and acetylsalicylic acid (ASA) (63%, n = 27), were most often the treatments of choice. Nine patients were given morphine, two patients received antacida and one patient was given a benzodiazepine.

Table 4 shows the physicians’ appraisal of the most probable cause of symptoms (“initial diagnosis”), and how they ended up treating the patient, including level of care. Half of the patients were admitted to hospital for further care, 86% (n = 43) because of suspected ischaemic heart disease. Musculoskeletal pain was the second most common cause of pain, managed in primary care (physician on-call or referred to GP) in 21 of the 22 patients (95%). Otherwise the patients were thought to have a variety of conditions, most of them managed at a primary care level. Of the 43 patients admitted to hospital with suspected ischaemic heart disease, 24 patients had NACA-scores between 4 and 6, indicating a severe illness.

**Table 4:** Initial diagnosis and level of care for treatment or follow-up with GP or specialist

	**Level of care for treatment or follow-up**				
	**Total**	**Managed at casualty clinic**	**Referred to GP**	**Referred to specialist non-urgently**	**Admitted to hospital**
**Appraisal of the most probable cause (“initial diagnosis”)**					
Ischaemic heart disease	**50**	2	3	2	43
Musculoskeletal pain	**22**	16	5	0	1
Psychiatric disease/anxiety	**12**	1	9	0	2
Pulmonary disease	**5**	3	1	0	1
Dyspepsia	**5**	1	4	0	0
Gastrointestinal disease, other than dyspepsia	**3**	1	0	0	2
Other diagnoses (arrhythmia, hypertensive crisis)	**3**	0	2	0	1
Total	**100**	**24**	**24**	**2**	**50**

## Discussion

We included 100 individual patients after interviews with 100 unique physicians, from a sample of 832 patients with chest pain. Median age of the included patients was 46 years, men constituted 58%. An ECG was taken in 92 of the patients, other laboratory tests in a majority. Of the 24 patients categorised to an acute level of response, two thirds had a NACA-score indicating a potentially or definitely life-threatening medical situation. Half of the patients had suspected ischaemic heart disease; the rest had a variety of conditions. Half of the patients were admitted to a hospital for further care, of which a large majority were thought to have heart disease.

A main strength of the study is the prospective registration of all patients with chest pain at the collaborating casualty clinics. To avoid dependency and an unbalanced weighting of the data; each patient and physician could only be included once. Answering of the questionnaire through telephone interviews enabled the interviewer to give precise instructions. We aimed to reduce recall bias by reaching the physicians shortly after the consultation, but some recall bias will be expected when interviewing a physician about a specific patient one or two days after an out-of-hour shift. The NACA-score has been widely used in studies concerning pre-hospital emergency medicine, and all included physicians were thoroughly explained how to use the scoring system. However, most of the interviewed physicians did not know the scoring system before the interview, and this might limit the reliability of its use. The data does not include the place of consultation (casualty clinic vs. ambulance), and the study design did not allow physician appraisal on how they decided the level of care for treatment. Due to resources available for interviews, the study was limited to 100 patients and doctors, a number that may limit the inclusion of more seldom diagnoses.

A recent study from Belgium [5] examined the initial diagnosis and referral rates in patients with chest pain in primary care. 37% of the patients received “heart disease” (26% “serious” and 11% “other”) as the initial diagnosis, while muscular disease accounted for 30% and somatoform disease 10%. Our results are comparable to these numbers, and also to other studies of chest pain in primary care [1,2,6], except our higher rate of suspected heart disease. In the 26% with “serious heart disease” [5], nearly half was admitted urgently to the emergency department, while a third was referred non-urgently to a specialist or the hospital. Our study showed that 43 of the 50 patients with suspected heart disease were admitted to hospital. An ECG was recorded in only 29% of the patients in the study from Belgium, which is considerably lower than in our study (92%). A prospective study from Norway investigating 1100 patients with acute chest pain assigned an acute response level (“red”), showed that 26% of the patients were in a life-threatening medical situation [3]. This number is equal to our study (26% with NACA-score 4–7), but our study includes patients with all three levels of response.

Patients with chest pain account for approximately 1-2% [1-4] of all consultations in primary care. Our study confirmed that ECG is the most important diagnostic tool in primary care. The high rate of ECG-testing might be explained by the fact that an ECG often is taken as a routine in patients with chest pain before they are examined by the treating physician. ECG is also readily available in all Norwegian casualty clinics, and most GP surgeries. Early ECG-testing is important in patients with severe illness suspicious of ischaemic heart disease, but it is also well known that over-testing, including use of ECG, and hospital admissions for chest pain can be unfortunate for patients suffering from anxiety or panic attacks. ECG is also still a diagnostic tool with limited sensitivity [10], and the test demands comprehensive knowledge in order to interpret the results in a reliable way.

Our study confirms that acute chest pain is a common diagnostic challenge in a primary care setting [1,2,5,6], and reflects much more than acute cardiac disease. However, the incidence of “heart disease” as the initial diagnosis in our study (50%) is higher than comparable studies. This may partly be explained by the study setting; patients at the casualty clinic are expected to have more acute and severe disease and higher prevalence of IHD than patients during daytime GP surgery hours [9]. On the other side, only 27 patients were given ASA, even though as many as 43 of the 50 patients with suspected heart disease, were admitted to a hospital. This suggests a lower probability of IHD in many of the patients, and few were given full “MONA”-treatment (morphine, oxygen, nitro-glycerine and ASA). The 50 patients with suspected IHD constituted most of the patients with a NACA-score ≥ 4. Still, even among the 43 patients with suspected IHD admitted to the hospital, almost half (19 of 43) had a NACA-score not indicative of a serious illness. In Norway, patients with chest pain in need of acute medical assistance are encouraged to call the national three digits emergency telephone number ″113″. A recent study from Norway [3], showed that in patients with chest pain handled by the emergency medical communication centres (EMCCs, responding to the ″113″ calls), 24% were brought directly to the hospital and managed by the ambulance staff alone, without involving the primary care physician on-call. Most ambulances in Norway can transmit an ECG to the hospital through telemedicine, and in many patients with acute chest pain the EMCC will “bypass” the casualty clinics. This might explain the low prevalence of patients given “MONA”-treatment at the casualty clinics in our study, but the 24% patients brought directly did nevertheless not have a NACA-score indicating a more severe illness [3].

The introduction of high-sensitivity (hs) troponin-tests, also in primary care, might change how GPs diagnose patients with acute chest pain in the near future. But it is important to bear in mind that an increased level of hs-troponin concentration alone does not give the diagnosis of acute myocardial infarction, according to recent guidelines [16]. Diagnosing chest pain in primary care is still a complex task because of the broad spectrum of causes, and it is important that a possible introduction of hs-troponin in primary care does not replace a comprehensive diagnostic approach.

Deciding the appropriate level of response can also be a difficult task, especially in patients with chest pain [3]. Our study showed that 63% of the patients with red response had a NACA-score indicating a potentially or definitely life-threatening medical situation, pointing to a certain degree of “*over*-triage”, well known to be resource demanding. On the other hand, 11 of the 76 patients (14%) given a yellow or green response level were also in need of rapid diagnostics and/or treatment (NACA ≥ 4), indicating possible “*under*-triage” and a potentially harmful underestimation of the patients’ severity of illness.

Half of the 100 patients in the study were admitted to hospital, and as many as 86% of the patients with an initial diagnosis of heart disease were admitted urgently. A recent study from the UK [17] showed that GPs in out-of-hours work with low “tolerance of risk” were more likely to admit patients to the hospital. Little is known about *how* physicians’ diagnose patients with chest pain in out-of-hours primary care and their reasons for deciding if the patient should be admitted to the hospital or not. More research is needed to elucidate this important part of GPs out-of-hours work.

## Conclusions

Patients with chest pain presenting at out-of-hours services in Norway are investigated for acute heart disease, but less than half are admitted to hospital for probable acute coronary syndrome, and only a minority is given emergency treatment for acute coronary syndrome. A wide variety of other diagnoses are suggested by the doctors for patients presenting with chest pain. Deciding the appropriate level of response for such patients is a difficult task, and both *over*- and *under*-triage probably occur in out-of-hours primary care.

## Competing interests

The authors declare that they have no competing interests.

## Authors’ contributions

RAB, EZ and SH planned and established the project, including the procedures for data collection, and designed the paper. RAB performed the analyses and drafted the first manuscript. All authors took part in rewriting and approved the final manuscript. All authors read and approved the final manuscript.

## Pre-publication history

The pre-publication history for this paper can be accessed here:

http://www.biomedcentral.com/1471-2296/15/51/prepub
